# Tumor-infiltrating lymphocytes in triple-negative breast cancer: molecular mechanisms, spatial regulation, and therapeutic implications

**DOI:** 10.1186/s13058-026-02285-w

**Published:** 2026-04-26

**Authors:** Yuefeng Li, Zhian Li, Jiangfeng Dai

**Affiliations:** Department of Surgical Oncology, Shaoxing Second Hospital, Affiliated to Shaoxing University, Shaoxing, 312000 Zhejiang China

**Keywords:** Triple, Negative breast cancer, Tumor, Infiltrating lymphocyte, Tumor immune microenvironment, Cancer, Associated fibroblast, Immunotherapy

## Abstract

Triple-negative breast cancer (TNBC) represents a profound therapeutic challenge due to its aggressive nature and limited treatment options. This review synthesizes recent advances in understanding the TNBC tumor immune microenvironment (TIME), moving beyond the simplistic “cold” tumor paradigm to depict it as a complex, integrated ecosystem. We detail how multilayered networks, including dynamic metabolic cross-talk, transcriptional reprogramming, cancer-associated fibroblasts (CAFs) heterogeneity, and spatial architecture, collectively shape antitumor immunity and determine the efficacy of tumor-infiltrating lymphocytes (TILs). While significant progress has been made, we critically examine the substantial translational gaps that remain, as many insights are still derived from preclinical models. We argue that rationally designed combinations, including the repurposing of clinically approved drugs, offer a near-term pragmatic pathway. Furthermore, we explore the conceptual shift from purely inhibitory strategies toward the selective overactivation of immune or metabolic pathways as a means to address current therapeutic challenges. Finally, we emphasize that the integration of high-dimensional data, including CAF taxonomy, immune phenotyping, and spatial mapping, is pivotal for the development of truly precision immunotherapies. This review concludes that the convergence of these approaches is forging a more comprehensive and actionable framework for the next generation of patient-tailored TNBC treatments.

## Background

Breast cancer is the most common malignancy in women, with a rising incidence in recent years [1]. Despite declining mortality, triple-negative breast cancer (TNBC) remains the subtype with the poorest prognosis [2]. Historically, systemic adjuvant therapy for TNBC has relied primarily on chemotherapy. [3, 4]. In recent years, the addition of immunotherapy to treatment regimens has been associated with enhanced remission and reduced recurrence risk in patients with TNBC [5, 6]. Among current approaches, immune checkpoint blockade targeting the programmed cell death protein 1 (PD-1) and its ligand PD-L1 (programmed death-ligand 1) represents the most widely used strategy [7]. These agents function by preventing the inhibitory interaction between PD-1 on activated T cells and PD-L1, which is frequently overexpressed on tumor and tumor-associated immune cells. Specifically, monoclonal antibodies with high-affinity binding domains selectively bind to PD-1 or PD-L1, thereby disrupting the signaling cascade that suppresses T-cell activation, cytokine production, and cytotoxic function. This blockade restores anti-tumor immunity and forms the mechanistic foundation for PD-1/PD-L1–based immunotherapy in TNBC [8, 9].

Currently, the clinical application and efficacy assessment of PD-1/PD-L1 immune checkpoint inhibitors are primarily guided by the Combined Positive Score (CPS), a quantitative metric that accounts for all PD-L1–positive tumor cells and immune cells, including tumor-infiltrating lymphocytes (TILs) [10, 11]. A CPS ≥ 10 has been identified as a critical threshold for clinical benefit from pembrolizumab plus chemotherapy in patients with metastatic TNBC, as evidenced by the KEYNOTE-355 trial, which demonstrated significantly improved progression-free survival (PFS) and overall survival (OS) in this subgroup [10]. However, this predictive value of CPS does not appear to extend to early-stage high-risk TNBC. In the KEYNOTE-522 trial, which evaluated pembrolizumab combined with neoadjuvant chemotherapy in patients with early-stage, high-risk TNBC, the addition of pembrolizumab significantly increased the pathological complete response (pCR) rate by 13.6% compared with placebo plus the same chemotherapy [12]. Given the lack of correlation between CPS and pCR, ongoing research is intensively exploring alternative predictive biomarkers of therapeutic efficacy, including TILs and other constituents of the tumor immune microenvironment (TIME) [13]. TILs, including CD8^+^ cytotoxic T cells, CD4^+^ helper T cells, and other immune subsets, accumulate around TNBC tumors and play a critical role in the tumor immune response through coordinated interactions and cytotoxic activity [14]. However, the therapeutic efficacy of immune checkpoint inhibitors in TNBC is influenced by multiple factors, including the quantity, quality, and heterogeneity of TIL infiltration, as well as the complex molecular landscape of the TIME [15–17]. Collectively, these factors drive immune evasion and therapeutic resistance, ultimately leading to adverse clinical outcomes in TNBC. [18, 19].

Therefore, elucidating the mechanisms of TILs-mediated immune responses is essential for optimizing immunotherapeutic strategies. This review summarizes recent advances in TILs-related research in TNBC, with a focus on the molecular factors influencing TILs activity within the tumor microenvironment, and discusses their underlying mechanisms and potential implications for clinical translation.

## Direct mechanisms regulating TIL infiltration in TNBC

Among the signaling pathways implicated in tumor immunity, the cGAS-STING-IFN-ISGs (cyclic GMP–AMP synthase – Stimulator of Interferon Genes – Interferon – Interferon-Stimulated Genes signaling) pathway represents a central mechanism [20]. Cytosolic accumulation of aberrant double-stranded DNA (dsDNA), often resulting from tumor-intrinsic genomic instability, is sensed by cGAS [21]. Upon binding dsDNA, cGAS undergoes conformational changes and dimerization, enabling its catalytic domain to convert adenosine triphosphate (ATP) and guanosine triphosphate (GTP) into the second messenger 2′3′-cyclic GMP–AMP (cGAMP) [22]. cGAMP subsequently binds to and activates STING, a transmembrane adaptor on the endoplasmic reticulum, which then translocates to the Golgi apparatus to recruit and activate TANK-binding kinase 1 (TBK1). Activated TBK1 phosphorylates interferon regulatory factor 3 (IRF3), promoting its dimerization and nuclear translocation, where it binds to promoter regions of interferon-β (IFN-β) and multiple interferon-stimulated genes (ISGs), including C-X-C motif chemokine ligands (e.g., CXCL9, CXCL10, CXCL11) and C–C motif chemokine ligands (e.g., CCL2, CCL4, CCL5), thereby orchestrating transcriptional activation [23–25].

Secreted interferon-β (IFN-β) further amplifies this response in neighboring cells through the JAK–STAT (Janus kinase–signal transducer and activator of transcription) signaling cascade: binding to the interferon-α/β receptor (IFNAR) activates receptor-associated tyrosine kinase 2 (TYK2) and Janus kinase 1 (JAK1), leading to phosphorylation of receptor cytoplasmic tails and recruitment of STAT1/STAT2 (signal transducer and activator of transcription 1/2) [26]. Phosphorylated STAT1/STAT2 form a complex with Interferon Regulatory Factor 9 (IRF9), known as ISGF3, which translocates into the nucleus to enhance ISG expression, establishing a positive feedback loop [27, 28]. Collectively, this signaling cascade facilitates the recruitment and activation of immune effectors, including CD8^+^ T cells, CD4^+^ T cells, and regulatory T cells (Tregs), thereby shaping the TIME [28, 29].

Clinical studies have observed that several key molecules within the cGAS-STING-IFN-ISGs pathway are highly expressed in TNBC and are correlated with genomic instability and the degree of immune cell infiltration [30]. Previous investigations have highlighted that TNBC harboring breast cancer susceptibility gene 1 (BRCA1) mutations exhibits particularly pronounced genomic instability [31]. The MYC proto-oncogene (MYC) is a well-established oncogene in breast cancer, encoding the transcription factor c-MYC [32, 33]. In BRCA1-deficient TNBC, the presence of genomic instability drives MYC overexpression, which is associated with poor clinical outcomes [30, 34]. Through TNBC cell-based assays and MYC-overexpressing mouse models, a recent study demonstrated in vitro and in vivo that MYC directly suppresses the cGAS–STING–IFN–ISG axis at the transcriptional level, thereby limiting TIL infiltration and fostering an “immune-excluded” phenotype. Mechanistically, MYC forms a co-repressive complex with Myc-interacting zinc finger protein 1 (MIZ1), which binds directly to the promoter regions of ISGs, suppressing their transcription. This inhibition disrupts the synthesis of key cytokines such as STAT1 and CXCL10—the downstream products of DNA damage-induced activation of the cGAS–STING pathway in BRCA1-deficient TNBC—ultimately impairing TIL recruitment. Notably, in mouse models, this process is unaffected by STING activation via vadimezan (DMXAA, a STING agonist), but can be reversed by the MYC V394D mutant, which relieves transcriptional repression of ISGs, thereby mitigating the suppression of TIL recruitment within the TIME. These findings suggest a potential therapeutic strategy to overcome the “cold” immune phenotype of BRCA1-deficient TNBC [34].

Several studies across different malignancies have demonstrated a close mechanistic relationship between MYC and DNA Methyltransferase 1 (DNMT1), a nuclear protein implicated in tumorigenesis [33, 35, 36]. In TNBC, DNMT1 expression has been shown to be transcriptionally upregulated under the influence of MYC, thereby modulating tumor immunity through the cGAS–STING–IFN–ISG pathway. Specifically, DNMT1 catalyzes cytosine methylation at the STING promoter, leading to hypermethylation and transcriptional silencing. This represses STING protein expression on the endoplasmic reticulum membrane, attenuates immune-related cytokine production, and diminishes TIL infiltration. Importantly, treatment with decitabine, a DNMT1 inhibitor and demethylating agent, was found to reverse MYC-driven STING promoter methylation, restore STING expression, and enhance CD8^+^ T-cell infiltration, effectively converting the immune landscape from “cold” to “hot.” Moreover, decitabine synergized with anti–PD-1 therapy in murine models, further augmenting CD8^+^ T-cell activity and antitumor efficacy [37].

Beyond MYC- and DNMT1-mediated transcriptional regulation of the cGAS–STING axis, phosphatase and tensin homolog (PTEN) loss, which is another tumor-intrinsic alteration that frequently co-occurs with MYC amplification in TNBC, adds an additional regulatory layer to this pathway [38].—PTEN, a classical tumor suppressor with both lipid and protein phosphatase activity, is commonly deleted in TNBC, leading to enhanced tumor invasiveness and metastatic potential [39]. Recent work has identified a downstream mechanism whereby PTEN controls post-translational turnover of STING via Ras-associated binding protein 7 (RAB7). RAB7, a small GTPase of the Ras superfamily, in its dephosphorylated state mediates the routing of STING on endosomal membranes toward lysosomal degradation [40]. Specifically, PTEN dephosphorylates RAB7 at serine 72, facilitating the lysosomal delivery and degradation of STING, whereas TBK1-mediated phosphorylation at the same residue restrains this process. Consequently, PTEN deficiency impairs STING degradation, resulting in its sustained accumulation and persistent induction of chemokines such as CXCL10, ultimately promoting TILs infiltration. Notably, PTEN-deficient TNBC exhibits marked sensitivity to STING agonists, and in vitro cell-based assays studies demonstrate that pharmacologic activation of STING significantly suppresses tumor cell proliferation while enhancing TILs migration. Collectively, these findings reveal a distinct therapeutic vulnerability in PTEN-loss TNBC and suggest that STING-targeted immunotherapy may represent a promising strategy for this molecular subtype [41].

**Fig. 1:**
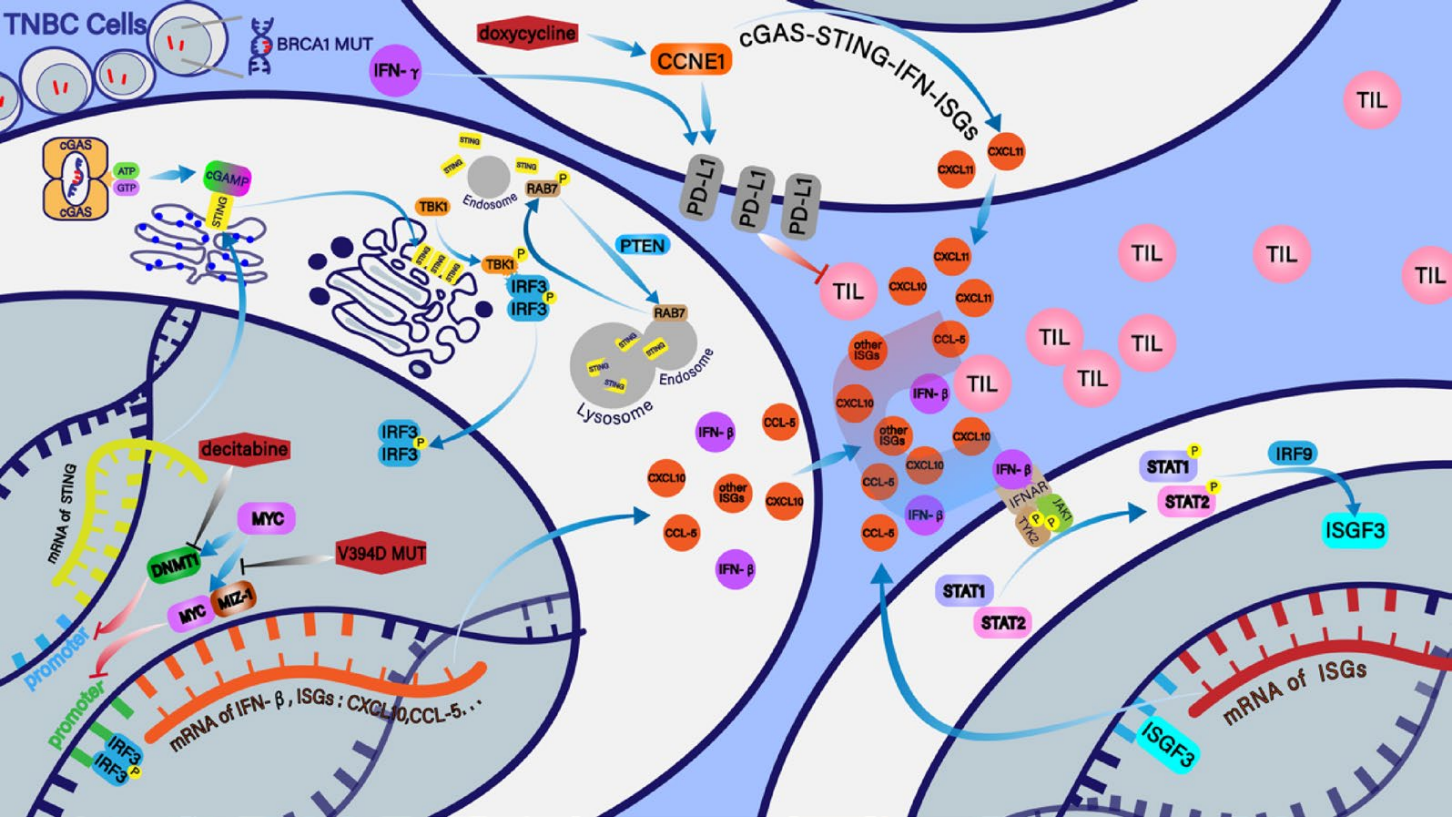
Tumor Cell–Driven Mechanisms Regulating TILs Infiltration in the TNBC Microenvironment. This schematic illustrates tumor-intrinsic pathways centered on cGAS–STING that regulate TIL recruitment and effector function in TNBC. Blue arrows indicate promotion, red T-shaped arrows indicate tumor-intrinsic inhibition, and black T-shaped arrows indicate inhibition by therapeutic interventions. Key molecular components are labeled directly in the figure; this legend focuses on the functional relationships and regulatory logic

CCNE1, an oncogene encoding Cyclin E1, has been shown through integrated analyses of TCGA and public datasets, as well as immune infiltration tools such as TIMER2.0, to be highly expressed in breast cancer, where its overexpression correlates with poor prognosis and altered immune infiltration [42, 43]. Comparative analyses of microarray datasets from GEO further revealed that CCNE1 expression is significantly higher in TNBC compared with non-TNBC, a finding subsequently validated by cellular experiments. Elevated CCNE1 expression was strongly associated with unfavorable prognosis in TNBC, suggesting its potential as a prognostic biomarker [44]. Recent studies, based on clinical samples and database analysis, have further validated through cell-based assays that CCNE1 amplification promotes genomic instability and activates the cGAS–STING–IFN–ISG axis in TNBC cell lines, thereby inducing ISG expression (e.g., CXCL9, CXCL11) and enhancing TIL recruitment. In addition, doxycycline-induced have shown that doxycycline-induced CCNE1 overexpression upregulates PD-L1 autonomously; importantly, when combined with IFN-γ stimulation, CCNE1 further synergized to amplify PD-L1 expression, thereby reinforcing an immunosuppressive program that facilitates TNBC immune evasion [45]. From a translational perspective, subgroup analysis of the I-SPY2 trial which evaluates the efficacy of neoadjuvant chemotherapy combined with immune checkpoint inhibitors in high-risk, early-stage TNBC, showed that patients achieving pathological complete response (pCR) had significantly higher CCNE1 copy numbers than non-pCR patients [45, 46]. Although the precise mechanistic underpinnings remain to be fully elucidated, these findings collectively support CCNE1 as a promising biomarker for predicting immunotherapy responsiveness and provide a rationale for developing CCNE1-informed combination strategies in TNBC. (Fig. 1).

Beyond tumor-intrinsic mechanisms, cancer-associated fibroblasts (CAFs) are also critical direct participants in shaping TIL-mediated antitumor immunity [47, 48]. CAFs are highly heterogeneous within the tumor microenvironment (TME) and have been closely linked to therapeutic resistance against immunotherapy in TNBC [49]. Multiple classification systems have been proposed to explore the immunological roles of CAF subtypes. Costa and colleagues stratified CAFs based on protein marker expression into four subtypes: CAF-S1, S2, S3, and S4. In treatment-naive TNBC surgical samples, CAF-S1 and CAF-S4 were found to be enriched, with CAF-S1 particularly associated with high levels of Treg infiltration. Using in vitro cell-based assays with confirmation in human tissue specimens, CAF-S4 lacked immunosuppressive activity, whereas CAF-S1 secreted CCL5 and other cytokines to recruit CD4^+^CD25^+^ T cells. CAF-S1 further promoted their differentiation into highly suppressive CD4^+^CD25^+^FOXP3^+^ Tregs via sustained interactions with tumor necrosis factor receptor superfamily member 4 (OX40) and PD-1 through its high expression of OX40 ligand (OX40L)and PD-L1, as well as through immunoregulatory molecules such as B7 homolog 3 protein (B7-H3, CD276), 5′-nucleotidase ecto (NT5E, CD73), and dipeptidyl peptidase 4 (DPP4, CD26). This immunosuppressive reprogramming contributed to a “cooled” immune microenvironment [15, 47, 50]. Building on these validations, the study proposed, from a translational perspective, DPP4 inhibitors, including sitagliptin, an FDA-approved drug for type 2 diabetes, as a potential therapeutic strategy for enhancing TNBC immunotherapy. Collectively, CAF-S1 may serve as a predictive marker of immunotherapy resistance, and the associated pathways provide a foundation for developing diverse new therapeutic targets [15, 51].

**Fig. 2:**
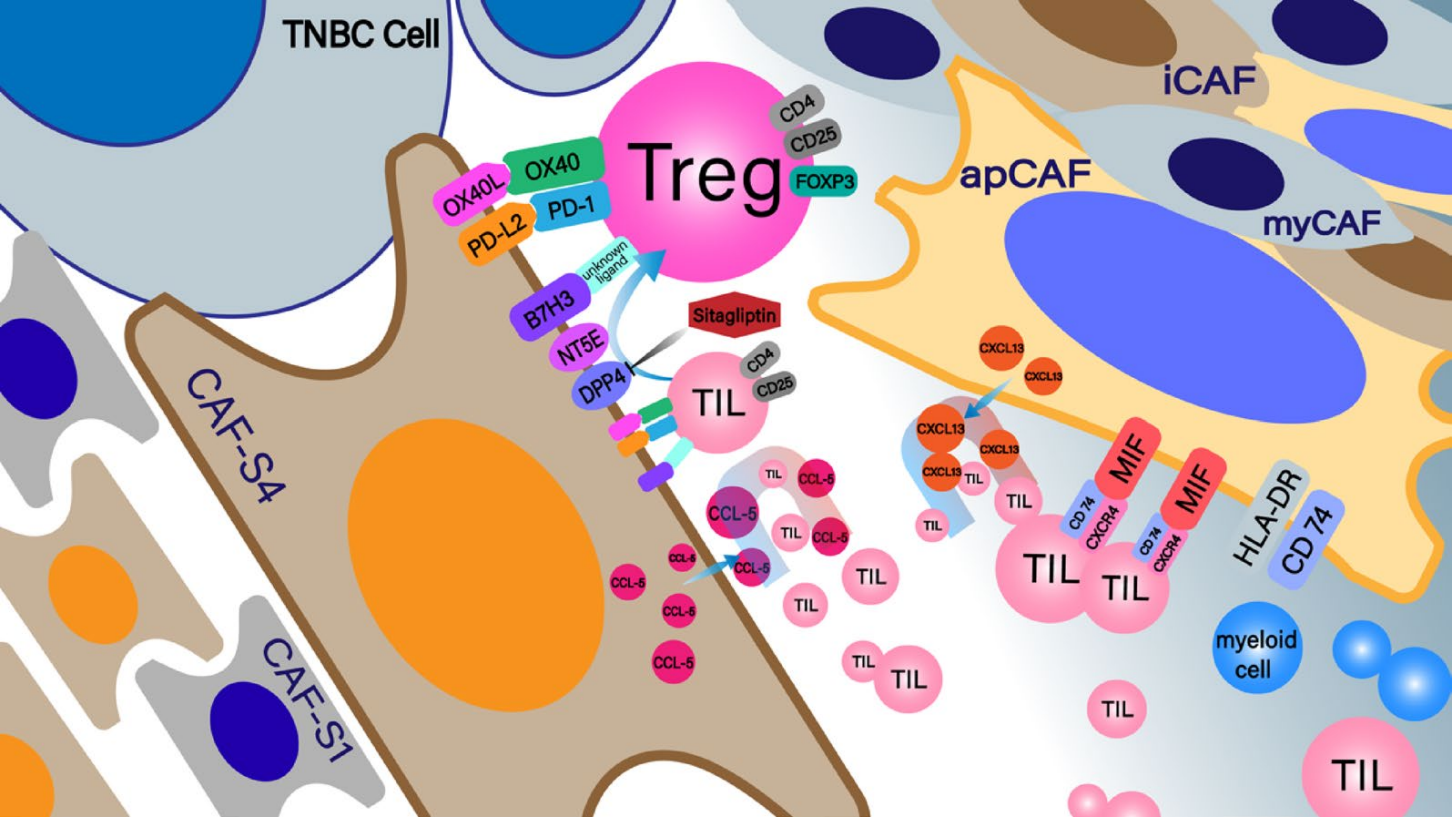
CAFs-Mediated Regulation of TILs Infiltration in the TNBC Microenvironment. This schematic illustrates how different CAF subsets in the TNBC tumor microenvironment regulate TIL recruitment, localization, and functional state. Blue arrows indicate promotion, while black T-shaped arrows indicate inhibition by therapeutic interventions. Key molecular components and mechanisms are labeled directly in the figure; this legend focuses on the functional relationships and regulatory logic mediated by CAFs

Alternatively, Tian and colleagues classified CAFs in TNBC specimens into three functional subtypes according to transcriptomic signatures: antigen-presenting CAFs (apCAFs), myofibroblastic CAFs (myCAFs), and inflammatory CAFs (iCAFs). apCAFs were characterized by high expression of major histocompatibility complex (MHC) class II molecules, including human leukocyte antigen–DR (HLA-DR), CD74, etc., conferring antigen-presenting capacity. CellChat analysis predicted robust immunosuppressive signaling between apCAFs and T cells or myeloid cells mediated through the acrophage migration inhibitory factor (MIF)–CD74/C-X-C chemokine receptor type 4 (CXCR4) axis, in which MIF engages the ligand–receptor complex formed by CD74 and CXCR4, consistent with their high expression profiles in apCAFs. Importantly, prognostic modeling revealed that TNBC patients with apCAF-associated gene expression signatures exhibited improved survival, higher CD8^+^ T-cell infiltration, and increased immune checkpoint expression [52]. Among these, C-X-C motif chemokine ligand 13(CXCL13) emerged as the most prognostically significant apCAF-associated protein. Gene set enrichment analysis (GSEA) further indicated that CXCL13 expression correlated positively with JAK–STAT pathway activation [52, 53]. Collectively, based on large-scale transcriptomic analyses with limited validation in clinical specimens, apCAF-based risk models may facilitate stratified assessment of immunotherapeutic outcomes in TNBC, while the characteristic expression of CXCL13 highlights a promising new direction for therapeutic target development. (Fig. 2).

## Indirect mechanisms regulating TIL infiltration in TNBC

Complex metabolic reprogramming is closely linked to the biological behavior of triple-negative breast cancer (TNBC) [54, 55]. Within the TME of TNBC, the Warburg effect—namely aerobic glycolysis—serves as the predominant pathway for tumor energy metabolism. [56]. Increasing evidence indicates that elevated glycolytic activity and lactate accumulation in the TNBC TME impair the antitumor function of TILs [57]. Among these factors, tumor-intrinsic glycolysis represents a critical determinant governing the capacity of TILs to participate in antitumor immunity. [58, 59].

TNBC cells frequently overexpress epidermal growth factor receptor (EGFR), a transmembrane protein that becomes activated upon binding epidermal growth factor (EGF). Activated EGFR interacts with pyruvate kinase M2 (PKM2), a key glycolytic enzyme, and phosphorylates the Y148 residue of PKM2, thereby inhibiting its enzymatic activity and blocking pyruvate production. In addition, EGFR activation downregulates miR-143, a microRNA that binds to the 3′-UTR of hexokinase 2 (HK2) mRNA, thus relieving miR-143–mediated suppression of HK2 expression and further enhancing glycolysis. Together, these dual mechanisms create a “glycolytic bottleneck,” leading to the accumulation of the intermediate phosphoenolpyruvate (PEP). PEP is subsequently reduced to lactate by lactate dehydrogenase (LDH) and exported into the extracellular space, resulting in a marked reduction in activated CD8^+^ T cells capable of producing interleukin-2 (IL-2) and Interferon gamma (IFN-γ). This metabolic shift diminishes antitumor immunity and promotes immune evasion. Notably, combined treatment with the EGFR tyrosine kinase inhibitor (TKI) gefitinib and 2-deoxy-D-glucose (2-DG), a glucose analog that competitively engages HK2 and disrupts its metabolic function, markedly suppresses TNBC progression, as demonstrated by in vitro experiments in TNBC cell lines and in vivo studies in mouse models, underscoring the therapeutic potential of exploiting metabolic vulnerabilities to augment antitumor efficacy. [60, 61].

Beyond HK2-dependent regulation, recent studies highlight lactate dehydrogenase A (LDHA) as another essential driver of lactate generation in TNBC. N-acetyltransferase 10 (NAT10) promotes LDHA expression by stabilizing JunB mRNA through ac4C modification; JunB acts as a transcription factor that enhances LDHA transcription. Increased LDHA activity accelerates lactate production and inhibits TIL infiltration. This pathway can be effectively reversed by Remodelin, a selective NAT10 inhibitor, which enhances TIL recruitment into the TME. In addition, Remodelin promotes the internalization of cytotoxic T-lymphocyte–associated protein 4 (CTLA-4), a key inhibitory receptor that attenuates T-cell activity into the endocytic recycling pathway, ultimately resulting in a relative reduction of CTLA-4 on the T-cell surface. These mechanisms were validated in vitro and in TNBC mouse models, which further demonstrated that combining Remodelin with anti–CTLA-4 antibodies (e.g., ipilimumab) markedly augments antitumor immunity in TNBC, yielding substantially enhanced therapeutic efficacy [62–64].

Recent research further reveals that tumor endothelial cells (ECs) participate in shaping TNBC immune responses during chemotherapy-induced immunogenic cell death (ICD). Changes in EC glycolysis contribute to immune remodeling and chemoresistance. A distinct immunosuppressive EC subset (ECs-sub2), which is enriched in paclitaxel-resistant TNBC, exhibits high expression of immune checkpoint molecules including PD-L1. ECs-sub2 are characterized by abundant surface expression of tumor necrosis factor receptor 2 (TNFR2), a transmembrane protein that, upon binding TNF-α in the TME, activates downstream NF-κB (nuclear factor kappa-light-chain-enhancer of activated B cells) signaling. Activated NF-κB translocates into the nucleus and binds to promoter regions of key glycolytic regulators, including glucose transporter 1 (GLUT1) and HK2, thereby suppressing their transcription and reducing EC glycolytic flux. This metabolic reprogramming exacerbates IFN-γ–induced PD-L1 upregulation on ECs, thereby intensifying their suppressive cross-talk with infiltrating CD8^+^ T cells within the TME. The amplified PD-L1–CD8^+^ T-cell engagement imposes a dominant immunosuppressive barrier that sharply restricts effective TIL activity. These findings, validated in murine TNBC models and patient tissue specimens, consequently support therapeutically disrupting TNFR2 signaling on ECs as a compelling strategy to restore endothelial metabolic fitness, reawaken TIL infiltration, and dismantle the immunosuppressive architecture that drives TNBC progression. [65, 66].

Dysregulated lipid and amino acid metabolism also contributes to immune remodeling in TNBC, although most existing studies have largely remained at the level of correlative associations among molecular factors [54, 57]. B7-H3, a recently recognized immune checkpoint highly expressed on tumor cells, has traditionally been investigated for its inhibitory effects on T-cell activation. Mechanistically, B7-H3 is proposed to engage an as-yet unidentified receptor on T cells, thereby suppressing NF-κB–dependent transcriptional programs essential for T-cell activation. Evidence from preclinical studies across multiple tumor types, including TNBC, indicates that this suppression leads to impaired transcription of IL-2 and IFN-γ, attenuation of PI3K/Akt/mTOR (phosphatidylinositol 3-kinase/protein kinase B/mechanistic target of rapamycin) signaling, and consequent reduction in aerobic glycolysis—collectively constraining the proliferative capacity and effector function of both CD4^+^ and CD8^+^ T cells and fostering an immunosuppressive tumor microenvironment [67, 68]. More recent mechanistic studies based on cellular experiments and in vivo validation in TNBC mouse models have expanded the functional repertoire of B7-H3 by uncovering its role in regulating antitumor immunity through lipid metabolic reprogramming. Upon downregulation of B7-H3, the PI3K/Akt/mTOR axis becomes reactivated, promoting the nuclear translocation of sterol regulatory element–binding protein 1 (SREBP1). Nuclear SREBP1 subsequently binds to the promoter region of fatty acid synthase (FASN), driving its transcription and enhancing de novo fatty acid synthesis. This lipid metabolic “compensation” serves as an adaptive survival mechanism that enables tumor cells to withstand immune pressure. On the basis of this mechanism, therapeutic strategies combining B7-H3 blockade which alleviates suppression of TIL activity with pharmacologic inhibition of FASN (e.g., HY-120394) effectively prevent tumor cells from exploiting this metabolic escape route. Such dual-targeting approaches markedly enhance TIL infiltration and effector function in TNBC models [69]. Collectively, these findings provide mechanistic insight into how lipid metabolic pathways intersect with immune checkpoint regulation to shape TIL-mediated antitumor immunity in TNBC.

**Fig. 3:**
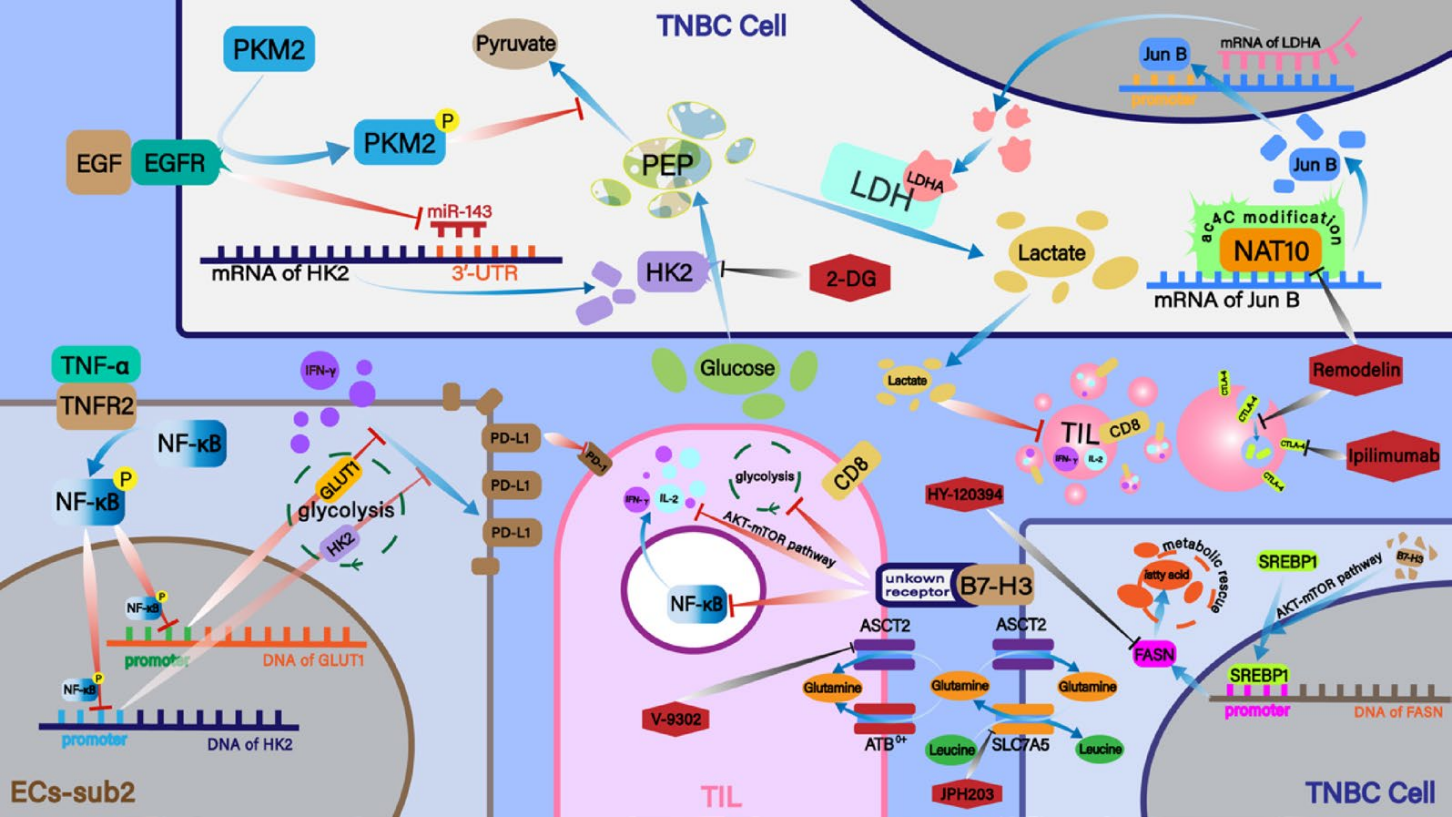
Multifaceted Metabolic Regulation of TILs Infiltration in TNBC. This schematic illustrates how tumor cells and tumor endothelial cells in the TNBC microenvironment regulate TIL recruitment and effector function through metabolic pathways. Blue arrows indicate promotion, red T-shaped arrows indicate inhibition, and black T-shaped arrows indicate inhibition by therapeutic interventions. Key molecular components are labeled directly in the figure; this legend focuses on the functional relationships and regulatory logic

In amino acid metabolism, most studies focus on glutamine dependency during TNBC metabolic reprogramming. TNBC exhibits “glutamine addiction,” enabled by high expression of the glutamine transporter alanine-serine-cysteine transporter 2 (ASCT2) and glutaminase (GLS), the enzyme that converts glutamine to glutamate for anabolic utilization. This metabolic configuration allows TNBC cells to uptake and consume large quantities of glutamine from the TME, thereby competitively suppressing the metabolic fitness and effector function of TILs. V-9302, a selective ASCT2 inhibitor, blocks glutamine uptake in TNBC cells via competitive inhibition. Meanwhile, CD8^+^ T cells retain activity through the neutral and cationic amino acid transporter B (0 +) (ATB⁰^+^), enabling them to bypass the inhibitory effect of V-9302. This mechanism is supported by robust in vitro and in vivo evidence [70]. Another branch of amino acid–related research focuses on solute carrier family member solute carrier family 7 member 5 (SLC7A5, LAT1), a transporter that functions opposite to ASCT2 by exporting glutamine while importing essential amino acids (EAAs), including leucine. SLC7A5 is highly expressed in TNBC, positively associated with aggressive tumor behavior, and inversely correlated with TIL infiltration. JPH203, a selective SLC7A5 inhibitor, markedly reduces intracellular EAA levels and increases TIL infiltration. Moreover, preclinical studies showed JPH203 synergizes with anti–PD-1 therapy, producing superior antitumor effects in vivo compared with monotherapy[71, 72]. Although the detailed mechanisms remain to be fully elucidated, earlier studies provide strong rationale indicating that tumor cells outcompete TILs for EAAs in the nutrient-limited TME, thereby impairing TIL activity [73, 74] (Fig. 3).

## Spatial effects of TILs in TNBC

As discussed earlier, the immune microenvironment of TNBC exhibits substantial spatial heterogeneity [75]. Beyond variations in immune-cell infiltration, the mechanisms that shape the spatial distribution of TILs also represent potential therapeutic entry points for improving immunotherapy in TNBC. Several studies have identified fibroblast growth factor receptor (FGFR), a membrane-associated protein as a potential therapeutic target associated with TNBC prognosis [76, 77]. Analyses of human TNBC tumor specimens revealed that in TNBC-associated CAFs, FGFR activation drives the MAPK/ERK (mitogen-activated protein kinase/extracellular signal–regulated kinase) signaling pathway, which promotes CAF proliferation and migration. These CAFs accumulate around tumor nests to form a physical barrier that restricts immune-cell infiltration. Meanwhile, FGFR signaling enhances CAF secretion of vascular cell adhesion molecule-1 (VCAM-1), a transmembrane glycoprotein that binds to very late antigen-4 (VLA-4) on immune cells. Through this interaction, CAFs adhere to CD8^+^ T cells and other lymphocytes within the TNBC tumor microenvironment, trapping T cells outside the tumor parenchyma and preventing their transition into functional TILs. These findings were further substantiated in TNBC mouse models, in which pharmacologic inhibition of FGFR disrupted CAF-mediated T-cell exclusion and improved immune-cell infiltration. This phenomenon, characterized by ineffective adhesion, can be alleviated by combined treatment with FGFR inhibitors and immune-modulating agents [78, 79].

Within the TNBC tumour microenvironment, the spatial distribution of myeloid-derived cells represents an important determinant of tumour immunity. Tumour-associated macrophages (TAMs) are among the most abundant immune populations in TNBC, with infiltration levels exceeding those observed in other breast cancer subtypes [80, 81]. The classical framework categorizes TAMs into M1-like antitumour and M2-like protumour phenotypes. In tumour-margin regions, M2 macrophages further remodel extracellular matrix components via matrix metalloproteinases (MMPs), forming dense stromal barriers that impede T-cell infiltration in cooperation with CAFs. In TNBC lesions, M1 macrophages promote TIL recruitment and activation through secretion of chemokines such as CXCL9 and CXCL10, driving Th1 polarization and antitumour responses. In contrast, M2 macrophages induce T-cell dysfunction through multiple mechanisms, including PD-L1–mediated contact inhibition, secretion of IL-10 and TGF-β to suppress Th1 and CD8^+^ T-cell activity, and production of CCL17 and CCL22 to recruit immunosuppressive Treg populations [82]. As insights into TAM biology across cancers continue to expand, traditional subtype-based frameworks are insufficient to explain the heterogeneous tumour immune landscape. Building on this understanding, recent TNBC studies have increasingly focused on identifying clinically relevant immunoregulatory molecular targets [83, 84].

**Fig. 4:**
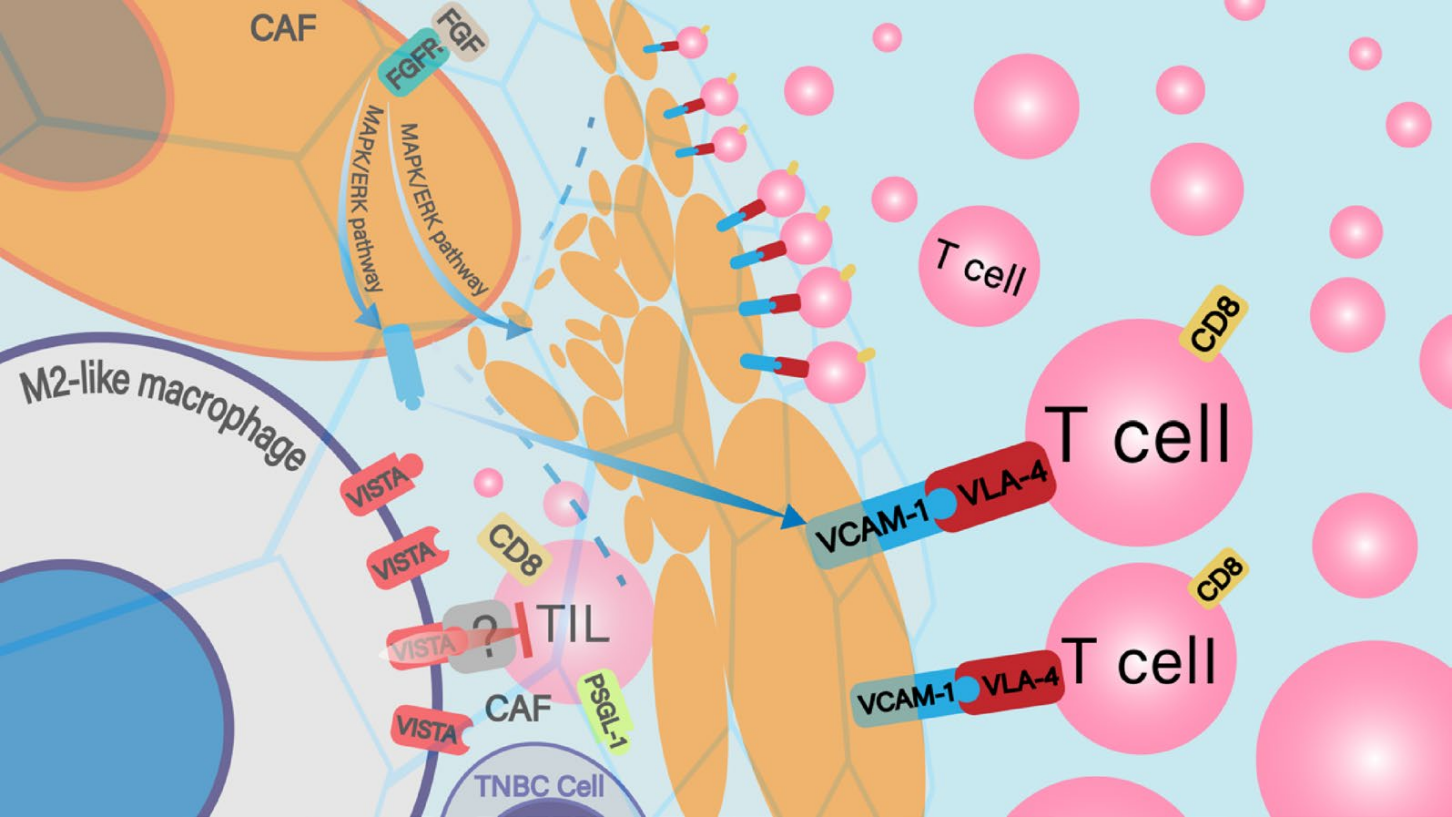
Spatial Organization and Functional Distribution of TILs in TNBC. This schematic illustrates the spatial organization of M2 macrophages and CAFs within the TNBC tumor microenvironment and their functional interactions with T cells across tumor core and margin regions. Blue arrows indicate molecular interaction or signalling pathways, while the red T-shaped arrow indicates inhibitory effect. Blue dashed lines illustrate the spatial clustering tendency of CAF populations within the tumour microenvironment. The blue hexagonal grid highlights the tumor margin area. Key molecular components and mechanisms are labeled directly in the figure; this legend emphasizes spatial and functional relationships influencing TIL infiltration and activity

V-domain Ig suppressor of T-cell activation (VISTA), a member of the immunoglobulin superfamily and mechanistically distinct from the PD-1/PD-L1 axis, is abundantly expressed on myeloid and granulocytic compartments, where it operates both as a ligand dampening T-cell activation and as a receptor conveying inhibitory signaling [85]. In TNBC, VISTA is particularly enriched on tumor-infiltrating M2-like macrophages, functioning as a dominant myeloid-derived immune checkpoint. Upon engaging infiltrating CD8^+^ T cells, VISTA transmits potent suppressive signals that blunt their cytotoxic program, resulting in marked reductions in effector molecules such as granzyme B (GZMB) and IFN-γ. As a consequence, TILs that physically penetrate the tumor parenchyma become functionally silenced, yielding a state of pseudo-enrichment in which numerical accumulation masks profound functional exhaustion and ultimately resulting in ineffective infiltration, synergistic with the outcome of intratumoural M2-like macrophage–mediated suppression described above. Importantly, preclinical evidence from TNBC mouse models demonstrated that this macrophage-driven suppressive axis can be alleviated by VISTA-blocking agents, underscoring its therapeutic relevance. Although the precise molecular interactions governing VISTA-mediated immunosuppression remain incompletely defined, current evidence suggests that its activity does not depend on P-selectin glycoprotein ligand-1 (PSGL-1). Elucidating alternative VISTA ligands, receptor partners, and downstream signaling modules will therefore be essential for fully characterizing this pathway and may open new avenues for rational immunotherapeutic strategies in TNBC [86, 87] (Fig. 4).

In addition, emerging technologies such as multiplex immunofluorescence, imaging mass cytometry, and spatial transcriptomics have enabled a more precise visualization of the spatial relationships between myeloid populations and TILs. Using these approaches, Moamin MR and colleagues observed that specific TAM subsets co-localize with Treg cells in perivascular niches of TNBC, forming three-cell clusters with CD4^+^ and CD8^+^ T cells. This spatial architecture was particularly evident after neoadjuvant chemotherapy, suggesting that perivascular regions represent key ecological niches in which TAMs regulate T-cell function [88]. Furthermore, Yang et al. applied spatial transcriptomics to demonstrate a potential spatial exclusion between myeloid-derived suppressor cells (MDSCs) and CD8^+^ T cells in titin (TTN)-deficient TNBC. Multiplex immunofluorescence confirmed enrichment of MDSCs around tumour cells and marginalization of CD8^+^ T cells in situ. Subsequent in vitro co-culture and in vivo functional assays indicated that this spatial pattern depends on delta-like ligand 4 (DLL4)-driven metabolic reprogramming of MDSCs through activation of Notch signalling, a pathway known to play key roles in vascular development and immune regulation [89]. Collectively, these technological advances provide important insights into how spatially organized myeloid regulation shapes antitumour immunity in TNBC.

These findings underscore the importance of incorporating myeloid-driven spatial regulation into therapeutic strategies aimed at improving immunotherapy responses in TNBC.

## Therapeutic implications and translational perspectives

Systemic treatment for TNBC remains largely chemotherapy-based. The incorporation of PD-1/PD-L1 inhibitors has marked a conceptual transition from purely cytotoxic tumour suppression toward strategies that actively engage and reinvigorate antitumour immunity [90]. Within this emerging framework, accumulating evidence indicates that multiple molecular classes may serve as strategic nodes for selective immune activation in TNBC. Tumour-intrinsic oncogenic pathways, exemplified by MYC-mediated suppression of the cGAS–STING axis, can be therapeutically relieved through epigenetic modulation or pathway-specific inhibition, thereby reactivating suppressed innate immune signalling and facilitating the conversion of immune-cold tumours into inflamed phenotypes.

In parallel, metabolic reprogramming within the tumour microenvironment represents another actionable layer. Interventions aimed at alleviating lactate accumulation, inhibiting aberrant fatty acid metabolism, or modulating amino acid utilization may reverse metabolic constraints on TIL infiltration and effector function. Such strategies move beyond checkpoint release and instead reshape the biochemical landscape that dictates immune competence.

Furthermore, targeting spatially defined suppressive pathways, including FGFR-driven stromal signalling and myeloid-enriched checkpoints such as VISTA, offers additional opportunities to dismantle immune-restrictive niches both at the tumour margin and within tumour cores. Collectively, these approaches illustrate an emerging therapeutic framework in TNBC: rather than relying solely on inhibitory blockade, selective activation and reprogramming of immune-regulatory circuits provide multiple intervention points to reinforce antitumour immunity and remodel the immune microenvironment.

Nevertheless, it should be acknowledged that the majority of these mechanistic insights have thus far been validated primarily in preclinical TNBC cell line systems and murine models. Translational advancement will therefore depend on multidimensional strategies that integrate biomarker selection, rational drug repurposing, novel combination design, and continued target discovery.

Molecular stratification may refine patient selection for immunotherapy. Alterations in genes such as CCNE1 and PTEN have been implicated in modulating immune responsiveness and may serve as predictive biomarkers for immune checkpoint blockade efficacy. Incorporating such molecular indicators into clinical decision-making could help identify subgroups more likely to benefit from immune-based interventions.

At the same time, rational combination strategies using existing agents provide a practical route toward clinical translation. Epigenetic modulation represents one such avenue. Although DNMT1 inhibitors such as decitabine have demonstrated limited efficacy as monotherapies in solid tumours, emerging early-phase studies, including trials combining DNMT1 inhibition with Poly(ADP-ribose) polymerase(PARP) inhibitors in advanced TNBC, suggest that epigenetic priming may sensitize tumours to subsequent therapeutic interventions [91]. These findings support further exploration of DNMT1 inhibitors in combination with immune checkpoint blockade, with careful evaluation of survival benefit and treatment-related toxicities.

Re-examining established drugs through the lens of metabolic and immune reprogramming may uncover novel combinatorial opportunities. For example, while the TKI gefitinib has shown limited benefit as monotherapy in TNBC, its combination with metabolic modulators 2-DG, as well as other strategies including NAT10i combined with CTLA-4 i or FASTi with B7-H3 blockade, suggests alternative strategies that exploit tumour metabolic vulnerabilities to enhance immune responsiveness [92].

Continued development of direct oncogenic pathway inhibitors remains essential. Although experimental models employing MYC variants with impaired DNA-binding capacity (e.g., V394D) have demonstrated that MYC-driven transcriptional programs can be functionally attenuated, translating these findings into clinically viable inhibitors remains challenging. Encouragingly, structure-informed efforts have led to the development of high-affinity MYC-binding molecules, such as Mizmetic, which disrupt MYC–DNA interactions and represent early pharmacological attempts to operationalize these mechanistic insights [93]. Nevertheless, further refinement in specificity, stability, and in vivo efficacy will be required before direct MYC inhibition can achieve clinical applicability. In addition, substantial challenges remain for the clinical translation of direct MYC inhibition, including the need to carefully evaluate pharmacological toxicity, off-target effects, and the impact of patient heterogeneity.

Finally, the exploration of emerging immune checkpoints and other surface targets continues to expand the therapeutic landscape. B7-H3, although widely studied across tumour types, exhibits varying functions across different cancer types and lacks a clearly defined receptor, posing a significant challenge for the targeted therapy of TNBC [94]. Recent development of bispecific antibody–drug conjugates(ADCs), such as DB-1419, targeting both B7-H3 and PD-L1, has demonstrated encouraging preclinical activity and favourable pharmacokinetic profiles, leading to early-phase clinical evaluation in advanced solid tumours, including TNBC (NCT06554795) [95].

**Fig. 5:**
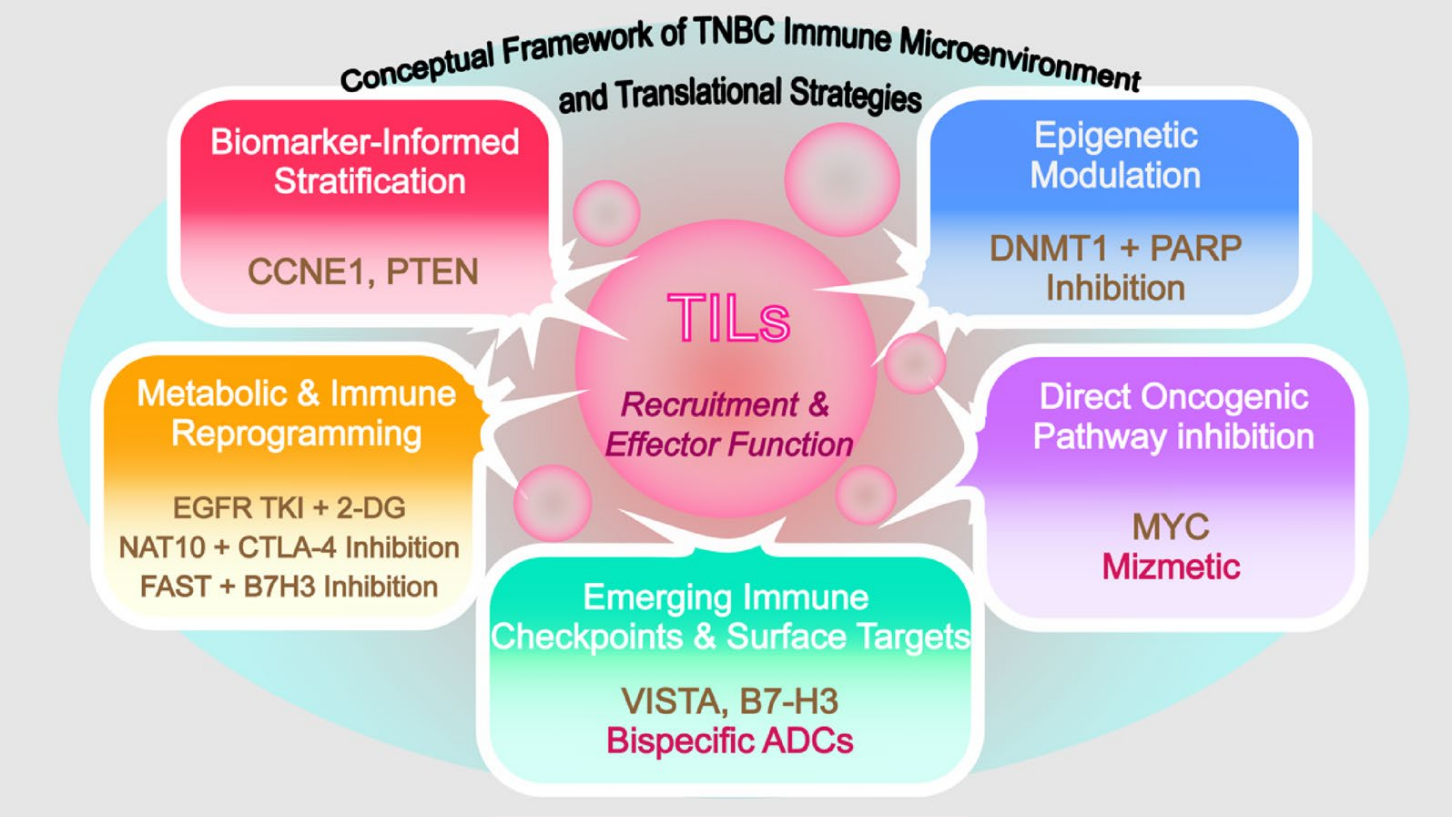
Conceptual framework of TNBC immune microenvironment and translational strategies. This schematic uses TIL recruitment and effector function as a central node around which major TNBC translational strategies are organized: biomarker-informed stratification (CCNE1, PTEN), epigenetic modulation (DNMT1 + PARP inhibition), metabolic and immune reprogramming (EGFR TKI + 2-DG, NAT10 + CTLA-4 inhibition, FAST + B7-H3 inhibition), direct oncogenic pathway inhibition (MYC, Mizmetic), and checkpoint/surface targets (VISTA, B7-H3, bispecific ADCs). The burst-style overlay of each module represents its effect on enhancing TIL recruitment and effector function, while the gradient background (outer light blue to inner light red) visually represents the transition of the tumour immune environment from “cold” to “hot.” The outermost layer summarizes collective translational strategies, including combination therapies and biomarker-guided approaches. Red-highlighted labels indicate representative emerging specific molecules or therapeutic agents. Mechanistic details for each module are presented in Figs. 1–4

Collectively, these translational directions illustrate that the future of TNBC immunotherapy lies not in single-pathway inhibition but in biomarker-informed, mechanism-driven combinatorial strategies that progressively convert mechanistic insight into clinically actionable interventions. (Fig. 5).

## Conclusion

The immunobiology of TNBC is governed by an intricately organized TME, in which tumour cells, CAF subsets, and diverse immune populations interact through dynamic metabolic, transcriptional, and spatial programs. These multilayered networks, rather than isolated signaling events, ultimately determine whether TILs can sustain effective antitumour activity. This systems-level perspective reframes TNBC not as an immunologically “cold” entity, but as a context-dependent ecosystem whose vulnerabilities can be strategically targeted.

Despite major advances, substantial translational gaps persist. Many current insights originate from reductionist or preclinical models, and only a fraction of promising targets have undergone rigorous clinical interrogation. Moreover, a considerable portion of existing evidence remains confined to molecular correlations, offering hypotheses about pathway involvement without definitive mechanistic validation. Deep mechanistic dissection, moving beyond associative findings, will therefore be essential for identifying actionable vulnerabilities and uncovering new avenues for TNBC immunotherapy. In the near term, rationally designed combinations, including repurposed agents with established safety profiles, may offer the most pragmatic route toward accelerating clinical impact.

Looking ahead, the field is advancing towards a shift in therapeutic strategies. Beyond inhibitory strategies, selective amplifing immune or metabolic circuits may reveal untapped antitumour potential. Integrating CAF taxonomy, high-dimensional immune phenotyping, and spatial mapping of stromal–immune interactions will be essential for developing truly precision immunotherapies. As these frameworks converge, they hold the potential to deliver more predictable, durable, and personalized treatment strategies for TNBC.

## Data Availability

No datasets were generated or analysed during the current study.

## Abbreviations

2-DG: 2-Deoxy-D-glucose
ACTB: Actin beta
APCs: Antigen-presenting cells
ASCT2: Alanine-serine-cysteine transporter 2
B7-H3: B7 homolog 3 protein
BRCA1: Breast cancer susceptibility gene 1
CAFs: Cancer-associated fibroblasts
CCL2: C–C motif chemokine ligand 2
CCL4: C–C motif chemokine ligand 4
CCL5: C–C motif chemokine ligand 5
cGAMP: 2′3′-Cyclic GMP–AMP
cGAS: Cyclic GMP–AMP synthase
CXCL9: C-X-C motif chemokine ligand 9
CXCL10: C-X-C motif chemokine ligand 10
CXCL11: C-X-C motif chemokine ligand 11
CXCL13: C-X-C motif chemokine ligand 13
CXCR4: C-X-C chemokine receptor type 4
CYP1A1: Cytochrome P450 family 1 subfamily A member 1
DLL4: Delta-like ligand 4
DNMT1: DNA methyltransferase 1
DPP4: Dipeptidyl peptidase 4
EGF: Epidermal growth factor
EGFR: Epidermal growth factor receptor
ERK: Extracellular signal-regulated kinase
FASN: Fatty acid synthase
FGFR: Fibroblast growth factor receptor
GZMB: Granzyme B
IFN-β: Interferon beta
IFN-γ: Interferon gamma
IL-2: Interleukin 2
IRF3: Interferon regulatory factor 3
IRF9: Interferon regulatory factor 9
ISGs: Interferon-stimulated genes
JAK1: Janus kinase 1
JAK-STAT: Janus kinase–signal transducer and activator of transcription
MAPK: Mitogen-activated protein kinase
MHC: Major histocompatibility complex
MIF: Macrophage migration inhibitory factor
MYC: MYC proto-oncogene
NF-κB: Nuclear Factor kappa-light-chain-enhancer of activated B cells
NT5E: 5′-Nucleotidase ecto
OX40: Tumor necrosis factor receptor superfamily member 4
OX40L: Tumor necrosis factor receptor superfamily member 4 ligand
PARP: Poly(ADP-ribose) polymerase
PD-1: Programmed cell death protein 1
PD-L1: Programmed death-ligand 1
PSGL-1: P-selectin glycoprotein ligand-1
PTEN: Phosphatase and tensin homolog
RAB7: Ras-associated binding protein 7
SLC7A5: Solute carrier family 7 member 5
STAT1/STAT2: Signal transducer and activator of transcription 1/2
SREBP1: Sterol regulatory element-binding protein 1
TBK1: TANK-binding kinase 1
TCR: T-cell receptor
TILs: Tumor-infiltrating lymphocytes
TIME: Tumor immune microenvironment
TKI: Tyrosine kinase inhibitor
TNBC: Triple-negative breast cancer
TTN: Titin
TYK2: Tyrosine kinase 2
VLA-4: Very late antigen 4
VCAM-1: Vascular cell adhesion molecule-1
